# Quantitative Research Designs, Hierarchy of Evidence and Validity

**DOI:** 10.1111/jpm.13135

**Published:** 2024-12-02

**Authors:** Paul Slater, Felicity Hasson

**Affiliations:** ^1^ Institute of Nursing and Health Research Ulster University Londonderry UK

**Keywords:** quantitative, research design, validity

## Abstract

This paper provides a summary of the main quantitative research designs. Quantitative research designs occur in a hierarchy of evidence, ranging from descriptive research designs such as cross‐sectional studies, and cohort designs, to more carefully constructed experimental designs like randomised control trials (RCT's). The quality of a study's findings is determined by factors affecting its internal validity and its application to other settings is gauged by its external validity.

## 1 Introduction

A research design is the framework used for the planning, implementation and analysis of a study. Quantitative research designs centre on numerical data collection and analysis to answer the research question or hypothesis. This is achieved through the application of objective, systematic and thorough processes to generate knowledge. These are reported in clear and transparent manner that permits replication of the methodology by other researchers. There are two main types of research design in quantitative research designs: descriptive (non‐experimental) and experimental. This paper provides an overview of each design.

## 2 Hierarchy of Evidence

**FIGURE 1:**
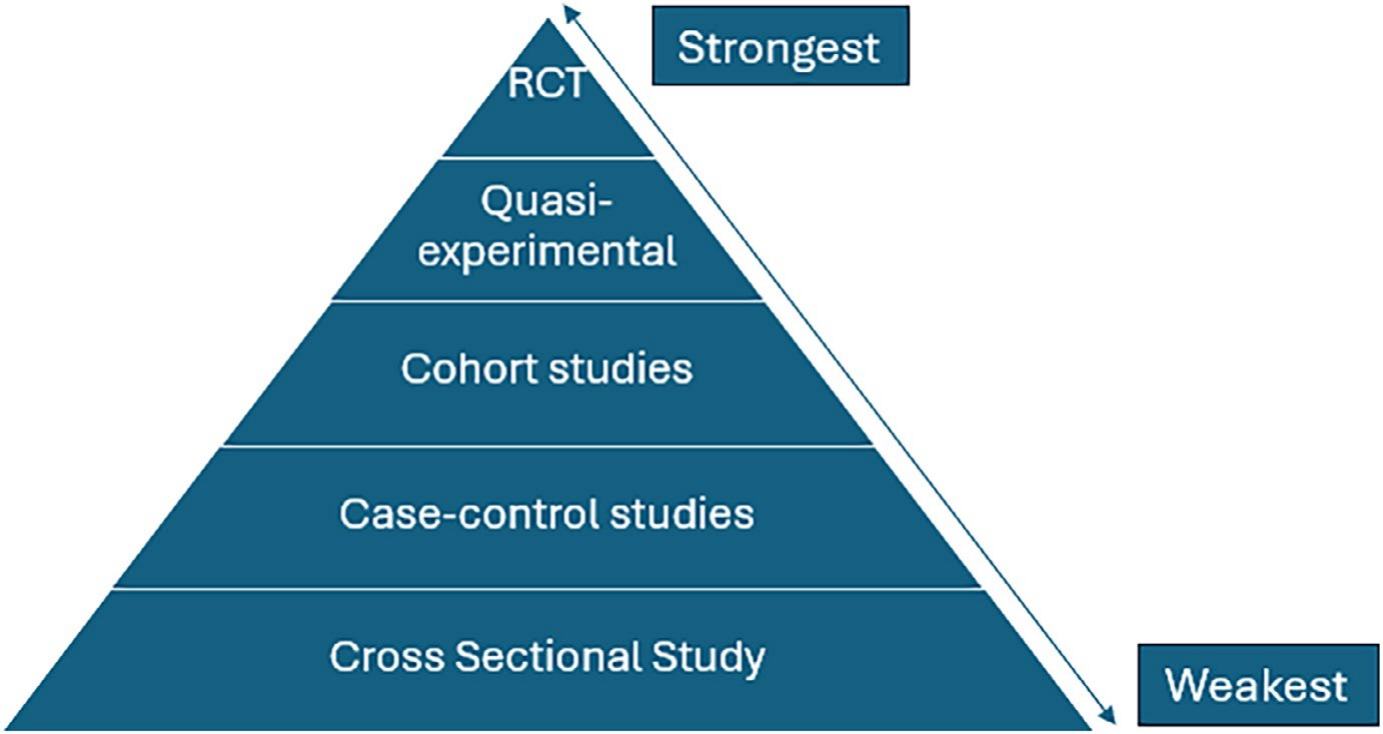
Hierarchy of evidence in quantitative research.

The hierarchy of evidence is used to rank the strength of results generated from research based on their internal validity (see Table 1). In essence, it illustrates that not all evidence is the same. Rather, there is an ‘evolution’ in research as more evidence is generated around a topic and this is reflected in the progression in research designs towards a Randomised Controlled Trial—‘the gold standard’. This is reflected in the hierarchy of evidence (see Figure 1). Descriptive research designs occupy the lower levels of the hierarchy of evidence, while experimental designs are graded on a higher level. This is due to the limitation to demonstrate causal effect between variables in the findings where relationships are simply correlation and as my old research methods lecturer used to repeat ‘correlation is not causation!’

**TABLE 1:** Threats to internal and external validity.

Information box
Definitions
Internal validity refers to the extent to which the results of a study are trustworthy and free from biases or errors, ensuring that the observed effects are truly due to the variables or interventions being studied, rather than external factors or confounding influences. It assesses whether a study accurately establishes a cause‐and‐effect relationship between the independent and dependent variables within the study's design.
External validity refers to the extent to which the results of a study can be generalised or applied to situations, settings, populations or times outside the study itself. In other words, it assesses whether the findings are relevant beyond the specific sample and conditions of the study.
When designing a study, the aim was to maximise both internal and external validity as much as possible, but this can be a challenge. Too controlled a study will undermine application to outside settings; too uncontrolled and the findings (causality) may be questionable.

Threat to internal validity	Definition	Impact
History	External events affecting the study.	Changes in outcomes may be caused by external factors, not the IV.
Instrumentation	Changes in measurement tools or procedures.	Inconsistent data collection affects comparability.
Selection bias	Systematic differences between groups before treatment.	Differences may be due to pre‐existing conditions. This should be checked statistically at baseline.
Attrition (mortality)	Participant dropout affects group representativeness.	Results may not generalise to the original population.
Testing effects	Influence of repeated testing on participant behaviour.	Improved performance due to familiarity with the test.
Maturation	Natural changes in participants over time.	Changes may be due to growth, learning or fatigue.

Threat to external validity	Definition
Sample characteristics (population validity)	The more diverse and representative a sample is, the more the findings of the study are generalisable to the wider population.
Setting characteristics (ecological validity)	Studies conducted in highly contrived or controlled environments, that is, laboratory work, limit applicability in the clinical field.

## 3 Descriptive Designs

In descriptive research, there are two broad categories of descriptive research, exploratory and descriptive. Exploratory designs are more flexible, have broad research questions and aim to uncover new insights. Descriptive designs are more structured and specific, have clearly defined research questions or hypotheses and while not setting out to establish causality, they do seek to provide a clear picture of the subject area.

The goal for descriptive designs was to provide insight into the characteristics or phenomenon of the population under investigation and does not try to establish a causal relationship. Therefore, they do not establish causality (threat to internal validity). Designs higher up the hierarchy of evidence may establish a temporal relationship (an event preceding to an outcome), but this should not be mistaken for causality. There are a variety of design types: cross‐sectional survey, case–control studies and cohort designs.

### 3.1 Cross‐Sectional Survey Design

This is one of the most common utilised research designs, as they are relatively inexpensive, convenient and can be used to use multiple factors and outcomes in one study. Unlike other types of observational studies, cross‐sectional studies do not follow individuals up over time, rather data are collected at a single point in time. As such they are commonly described as taking a ‘snapshot’ of the population. They may be based on an entire population or on a sample of the relevant population. A descriptive cross‐sectional design is the simplest form of cross‐sectional design which generally provides rudimentary information such as prevalence of health outcomes/symptoms, their impact and describe features of a population. Therefore, the variability within socio‐demographic variables provides useful information for future exploration. Its aims to provide information to better understand a phenomenon and to inform further research going forward. It is the transition between listening about the phenomenon (qualitative methods) to gaining a wider population‐based understanding of the phenomenon.

*A Working Example*: a cross‐sectional survey of Finnish psychiatric nurses (*n*=142) attitudes towards violence risk assessments using a standardised assessment tool. The instrument was sent electronically to a convenience sample of psychiatric nurses across 26 adult psychiatric inpatient wards in eight hospitals (Varpula, Ameel, and Lantta 2024).

The study details were reported using the STROBE guidelines (von Elm et al. 2007). These guidelines form a series of reporting guidelines for both quantitative and qualitive study designs contained in the ‘Enhancing the Quality and Transparency of Health Research (EQUATOR network https://www.equator‐network.org). This is an excellent resource for guiding the development of a research study, writing and publishing of high‐quality health research.

Correlation cross‐sectional surveys incorporate two variables, usually in the form of different questionnaires that the researchers suspect influence each other. In such cases, the researcher administers both questionnaires at the same time with the same participants and examines the relationship between the findings accordingly. This design provides more than a description of the patterns and trends, instead providing comparative findings of how variables are (not) related to each other.

### 3.2 Series Cross‐Sectional Survey Design

**FIGURE 2:**
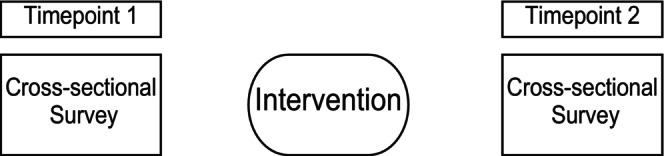
Series cross‐sectional design.

A series cross‐sectional survey design refers to when two or more cross‐sectional surveys are conducted within the same population at two or more timepoints, usually either pre and post an intervention or initiative (called bookshelf ending). The same survey is administered to the same population at multiple timepoints. It is a form of repeated measures; however, participants are not pair matched, across timepoints due to a new sample being drawn on each occasion (see Figure 2). While this a simple and popular method for measuring change within limited populations, such as schools or workplaces, the lack of repeated measures collected weakens the impact of the inferences that may be drawn from the findings.

### 3.3 Case–Control Studies

A case–control study is a type of observational study used to examine factors that may be associated with a particular outcome (disease or condition). Commonly used in epidemiology studies, it is particularly useful when investigating rare diseases/conditions. It begins with the outcome and retrospectively seeks to identify potential ‘risk factors’. As the name suggests, participants are split into two groups: cases—individuals who have the outcome of interest, and controls—matched individuals who do not have the disease or condition. A control group, who are matched to the case individuals, but do not have the outcome of interest are established to identify if the exposure(s) is/are found more commonly in the cases than the controls. Case–control approach is more feasible than experiments in which an outcome takes years to develop and enabling the examination of multiple risk factors at once.

*A Working Example*: An example of this research design in Kuipers et al. (2022) research paper comparing oral health related quality of life with patients with first episode psychosis against people from the general population. In the study participants were matched on age, gender and education. A 1:2 ratio of case versus comparison was set up. Cases were identified from clinical services according to DSM 5 (American Psychiatric Association 2013) and all participants were assessed using a standardised instrument.

### 3.4 Cohort (Prospective and Retrospective)

**FIGURE 3:**
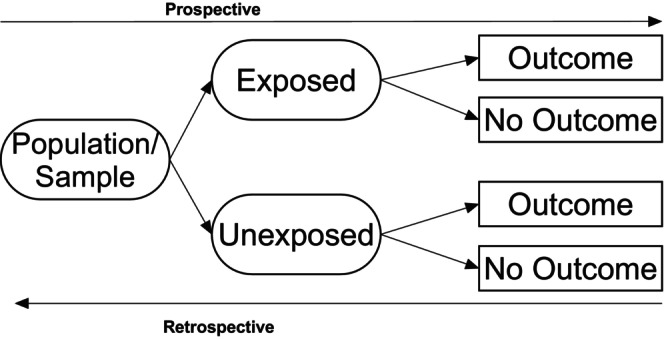
Cohort designs (Prospective and Retrospective).

Cohort designs are used to provide an understanding of the causes (temporal associations) of health outcomes or events. It is a longitudinal study design looking into future (prospective) condition development or the (retrospective) events that may have raised the odds of developing a condition (see Figure 3).

#### 3.4.1 Prospective Cohort Study

In a prospective cohort design, the sample is identified according to a specific criterion and subsequently coded as belonging to either the exposed or unexposed group. Participants are then followed over time at predetermined timepoints to examine the development or not of a condition or outcome. The evidence produced does not necessarily ensure causality but rather a temporal relationship between events. The onus is on the researcher to be sure that they are assessing the correct variables for predicting the outcomes from the outset. The design requires access to large sample sizes to accommodate follow‐up and participant attrition, to ensure statistically relevant outcome.

#### 3.4.2 Retrospective Cohort Study

This study is a review of previous existing data to examine predictors associated with events or outcomes (or not). A sample with, and without, a particular condition or outcome is identified, and historical datasets such as health records are explored to gain a fuller understanding of significant predictors of the outcome measure. This study design is relatively quick and inexpensive to run but can be limited to only looking at those variables that are available. As a research design, it is growing in popularity with the rise in big datasets and data linkage.

## 4 Experimental Designs

Experimental research designs contain a variety of elements designed to increase causality. This is achieved through randomisation to participants, manipulation of an independent variable, usually the intervention and the maintenance of strict controls of all other variables.

### 4.1 Quasi‐Experimental Design

It is not always feasible to satisfy all the conditions required of a randomised controlled trial due to ethical and/or practical issues that place natural constrains. However, we may wish to test interventions within these natural settings irrespective to the threat to internal and external validity. In quasi‐experimental research designs an element, such as a control group or randomisation, is forfeited but an intervention is implemented. This given rise to the two mains forms of quasi‐experimental design: the non‐equivalent groups design and the time‐series design.

*A Working Example*: In a study examining the effectiveness of spiritual adaptation of CBT in improving resilience, self‐esteem with opium use disorder patients in Egypt (Sonbol et al. 2024). This quasi‐experimental design included a non‐randomised control group, and a pretest‐posttest design assessed using standardised instruments, to look at the impact of a CBT intervention (vs. standard care) with Opium‐use Disorder.

#### 4.1.1 Non‐Equivalent Group Design

A non‐equivalent group design is similar to a classic RCT design, except randomisation is not performed, and as a consequence, we cannot be sure that participants' characteristics are not attributable as the cause of any effect change. Measures are collected as per a randomised controlled trial. An example of such a study is McCormack et al. (2007) where 12 wards in an acute hospital setting volunteered to be part of a study to implement person‐centred practices as a working model. Due to having a limited number of facilitators to provide the intervention, nine wards were assigned to intervention and three to control (waiting list). Data were collected pre and post intervention and results compared across timepoints and groups.

#### 4.1.2 Time‐Series Design

Participants in the experimental group are measured on multiple occasions on the same dependent variable before and after exposure to some treatment (interrupted time series design). Multiple measures before the intervention establish a stable baseline score prior to the intervention and likewise after the intervention, demonstrating impact and sustainability. If a control group is included (interrupted time series design with control group) in the design, this provides greater strength to the finding's validity.

### 4.2 Random Control Trail (RCT Design)

**FIGURE 4:**
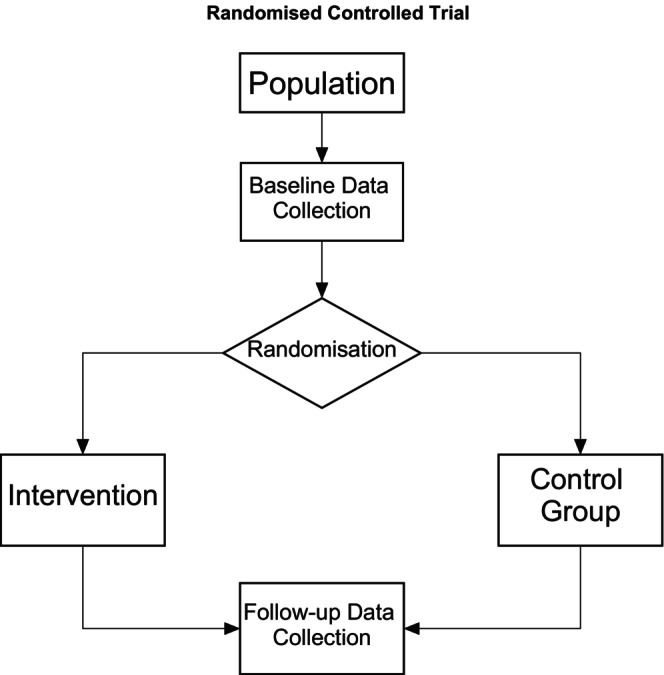
Basic elements of a Randomised Controlled Trial.

A randomised control trial is the gold standard in scientific evidence for evaluating the effectiveness of an intervention. It is a clinical trial, comparing a new or novel method or treatment being compared to existing standards of care that form two groups (intervention and control group). Randomisation—the sporadic allocation of participants to either a control or intervention arm of a study; controlled in that all other possible confounding variables are controlled for by the researcher. A population is identified, and a sample size calculated that provides sufficient respondents in both a control and intervention group with sufficient excess to cover any dropout. Baseline data are collected from all participants prior to randomisation. Randomisation aims to allocate participants without prejudice to either group. If the randomisation process is effective, there should be no significant differences on baseline scores across the two groups and this can be established statistically. The intervention is delivered while the control group receives treatment as usual. All efforts are made to deliver the intervention as documented (Fidelity). Data are collected post intervention and pair‐matched across individuals both pre and post. Statistical tests are used to examine if there is a difference between scores pre and post in the intervention and control group. Ideally an effective intervention will produce a positive change in scores in the intervention group and the control group score will remain relatively stable across timepoints (see Figure 4).

## 5 Internal and External Validity

Internal and external validity are key concepts (see Table 1) in quantitative research design and analysis. They are particularly relevant to experimental research design such as quasi experimental and RCT's. They are critical in determining the quality and applicability of research findings. Reporting tools such as the STROBE guidelines (von Elm et al. 2007) and checklist help researchers maximise the strength of a study, set within the context of the study and achieving study aims. Their application should be considered before commencing a study and not simply for reporting after completion.

The balance between internal and external validity may conflict with each other. Sometimes the pursuit to establish and control aspects of a study may ensure internal validity but to a point of being too contrived and artificial to generalise to the dynamic clinical settings. Therefore, a compromise needs to be achieved.

## 6 Conclusion

Quantitative research central tenant is objectivity which permits the researcher to generalise findings beyond a particular situation or setting. This paper outlined two overarching designs in quantitative research, descriptive (non‐experimental) and experimental. Descriptive research comprises designs were there is no manipulation of variables in the study. Experimental research designs generally share several commonalities such as randomisation, an independent variable that can be manipulated and a common dependent variable that can be measured in all groups in the study. The design type selected is primarily dependent on the aim of the investigation. Irrespective of the research design used, the researcher should be cognisant of maximising internal and external validity.

## Ethics Statement

The authors have nothing to report.

## Conflicts of Interest

The authors declare no conflicts of interest.

## Data Availability

The authors have nothing to report.
